# Insights in 17β-HSD1 Enzyme Kinetics and Ligand Binding by Dynamic Motion Investigation

**DOI:** 10.1371/journal.pone.0012026

**Published:** 2010-08-10

**Authors:** Matthias Negri, Maurizio Recanatini, Rolf W. Hartmann

**Affiliations:** 1 Pharmaceutical and Medicinal Chemistry, Saarland University, Saarbrücken, Germany; 2 Helmholtz Institute for Pharmaceutical Research Saarland, Saarbrücken, Germany; 3 Department of Pharmaceutical Sciences, University of Bologna, Bologna, Italy; The Scripps Research Institute, United States of America

## Abstract

**Background:**

Bisubstrate enzymes, such as 17β-hydroxysteroid dehydrogenase type 1 (17β-HSD1), exist in solution as an ensemble of conformations. 17β-HSD1 catalyzes the last step of the biosynthesis of estradiol and, thus, it is a potentially attractive target for breast cancer treatment.

**Methodology/Principal Findings:**

To elucidate the conformational transitions of its catalytic cycle, a structural analysis of all available crystal structures was performed and representative conformations were assigned to each step of the putative kinetic mechanism. To cover most of the conformational space, all-atom molecular dynamic simulations were performed using the four crystallographic structures best describing apoform, opened, occluded and closed state of 17β-HSD1 as starting structures. With three of them, binary and ternary complexes were built with NADPH and NADPH-estrone, respectively, while two were investigated as apoform. Free energy calculations were performed in order to judge more accurately which of the MD complexes describes a specific kinetic step.

**Conclusions/Significance:**

Remarkably, the analysis of the eight long range trajectories resulting from this multi-trajectory/-complex approach revealed an essential role played by the backbone and side chain motions, especially of the βFαG′-loop, in cofactor and substrate binding. Thus, a selected-fit mechanism is suggested for 17β-HSD1, where ligand-binding induced concerted motions of the FG-segment and the C-terminal part guide the enzyme along its preferred catalytic pathway. Overall, we could assign different enzyme conformations to the five steps of the random bi-bi kinetic cycle of 17β-HSD1 and we could postulate a preferred pathway for it. This study lays the basis for more-targeted biochemical studies on 17β-HSD1, as well as for the design of specific inhibitors of this enzyme. Moreover, it provides a useful guideline for other enzymes, also characterized by a rigid core and a flexible region directing their catalysis.

## Introduction

Cytoplasmic proteins are present in solution as an ensemble of conformations, which are in a dynamic equilibrium, strongly influenced by the presence of ligands (in case of enzymes: cofactors, substrates, inhibitors, or other proteins). This is especially true for enzymes following a bisubstrate kinetic, like *Escherichia coli* dihydrofolate reductase (DHFR) [1] and *human* 17β-hydroxysteroid dehydrogenase type 1 (17β-HSD1 or SDR28C1, according to the new nomenclature [2]; E.C. 1.1.1.62), where concerted dynamic motions are necessary between the enzyme conformations responsible for specific kinetic steps. An in-depth knowledge of both protein dynamic and its influence on ligand binding could effectively speed up rational drug design [1]. These new drugs might act not only by competing with the substrate for its binding site, but also by inducing a dynamic dysfunction of the enzyme by hindering the switch between its conformations [3].

**Figure 1:**
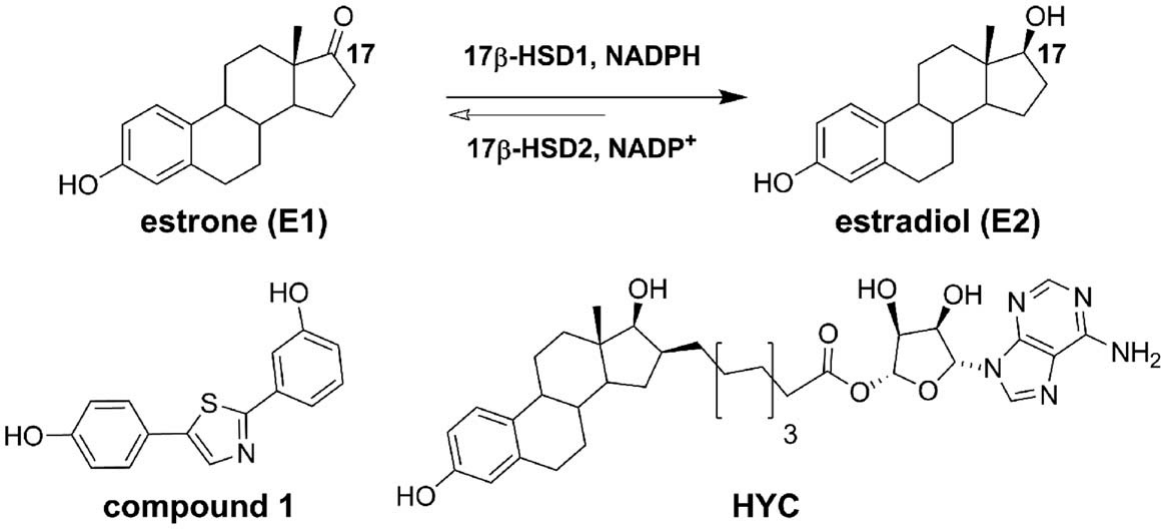
Reduction from E1 to E2 and non-steroidal and steroidal inhibitors. The substrate E1 is colored in blue, the cofactor NADPH in red. Amino acids, which are either involved in the catalysis or are responsible for ligand/cofactor stabilization, are colored in black and residue labelled. The hydrogen bonds are in dashed lines while the proton transfers are highlighted with arrows.

In estrogen target cells 17β-HSD1 catalyzes the NADPH-dependent reduction of estrone (E1) to the biologically highly potent 17β-estradiol (E2) [4]–[6] (Figure 1). It has been shown that in post-menopausal women with hormone-dependent breast cancer tumor proliferation is driven by increased levels of E2 [7]–[8]. As 17β-HSD1 is often overexpressed in breast tumor cells, it is considered as a novel therapeutic target [9]–[12].

**Figure 2:**
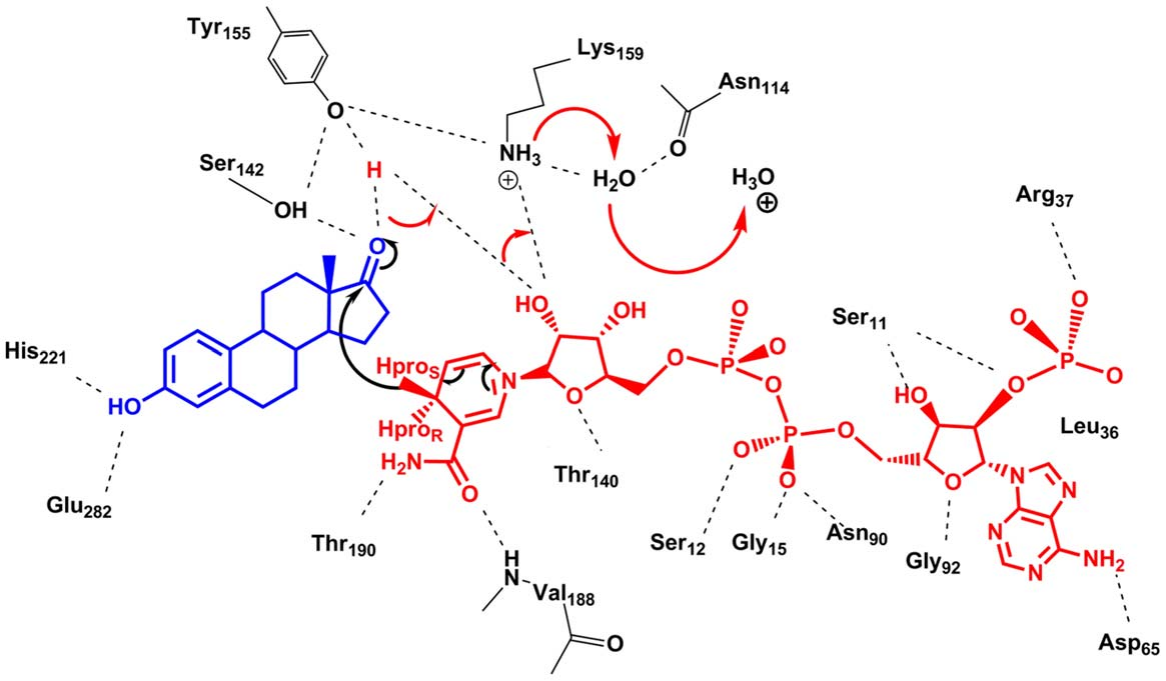
Postulated catalytic mechanism of 17β-HSD1. The substrate E1 is represented in blue, the cofactor NADPH in red. The amino acids, which are either involved in the catalysis or are responsible for ligand/cofactor stabilization, are colored in black and residue labelled. The hydrogen bonds are in dashed lines while the proton transfers are highlighted with arrows.

Recently, Cooper et al. elucidated the complete kinetic mechanism for the rat liver 3α-HSD and could assign different enzyme forms to the specific reaction coordinates [13]. 3α-HSD and nearly all other HSD enzymes are described to follow a sequential ordered bi-bi kinetic mechanism, where the cofactor enters first and exits last. The kinetic mechanism of 17β-HSD1 is still not fully clarified, although it has been reported to follow a rapid equilibrium random bi-bi mechanism (Figure S1), a peculiarity compared to other HSDs [14]–[16]. The high NADPH/NADP^+^ gradient (>500∶1) and the excess of NADPH with respect to E1 in vivo, as well as the thermodynamically favoured NADPH oxidation [17]–[18], suggest the presence of NADPH in the enzyme prior to steroid binding. Asn114, Ser142, Tyr155 and Lys159 form the catalytic tetrad of 17β-HSD1 [19], conserved in many HSD enzymes, and are involved in the *pro*-S hydride transfer from NADPH to the α-face of the C17 carbon as well as in the proton transfer between the OH group of Tyr155 and the C17 oxygen of E1 [19] (Figure 2).

Remarkably, the catalytic reaction of 17β-HSD1 is reversible *in vitro*; but it is unidirectional *in vivo*, because of the abundance of NADPH. No crystal structure of the ternary complex enzyme (E)-NADPH-E1 exists. We think that possible reasons for that might be problems in the crystallization process, i.e. low electron density for E1, but also the very fast transfer rate observed for the reduction of E1 (k_cat_ 1.5 s^−1^ for dimer [20]; 2.9 s^−1^ [21]). E2 has a two order of magnitude reduced binding affinity to 17β-HSD1 (K_M_ 4.6 µM) compared to that of E1 (K_M_ 0.03 µM), but a similar (less reduced) k_cat_ of 2.0 s^−1^ [21]. However, discordant k_cat_ and K_M_ values are reported in literature for both E1 and E2, in part depending on the cofactor used for the experiment (NADPH or NADH), its concentration (20 or 50 µM), etc. [14]–[15], [18], [20]–[22] The k_cat_ and K_M_ values reported here were obtained using NADP(H). Due to the excess of NADPH over NADP^+^ and due to the marked specificity of 17β-HSD1 for the E1 reduction using NADPH instead of NADP^+^ as cofactor (about 240-fold higher than for E2 oxidation) [21], we prospect that *in vivo* the backward reaction E2 to E1 does not take place. Moreover, the K_M_ of E1 is 12-fold lower when using NADPH (0.03 µM) than when using NADH (0.36 µM), hence underlining the crucial role played by the third phosphate group of the cofactor in the E1 reduction [21].

Steroidal and non-steroidal inhibitors of 17β-HSD1 have been reported (Figure 1) [9]–[11], [23]–[29]. The former mimic the substrate and are supposed to bind in the substrate binding site (SUB), a narrow hydrophobic tunnel [19]. They are stabilized by hydrogen bonds with Tyr155/Ser142 and His221/Glu282, located on both ends of the pocket, and, further, by hydrophobic contacts [19] Recently, Poirier et al. published a series of hybrid inhibitors, based on a E1/E2 core with substituents of various lengths in C16 position [26]. These inhibitors occupy both the SUB and the COF (cofactor binding site), competing with E1 and NADPH, as demonstrated by the binary complex E-HYC (PDB entry 1i5r). Up to now no crystallographic data exists describing the binding mode of non-steroidal inhibitors, although computational studies performed by various groups suggested some classes of non-steroidals to bind like their steroidal analogues in the SUB [9]–[11], [27]–[29] and others to occupy only partially the SUB protruding into the COF, like compound **1** [25].

Herein, we present the first, to our knowledge, computational investigation of the kinetic mechanism of 17β-HSD1, one of the thirteen 17β-HSDs belonging to the short-chain dehydrogenases/reductases (SDR), a large superfamily of NAD(P)H-dependent enzymes. Furthermore, the linkage between representative enzyme conformations and the five steps described for the random bi-bi mechanism, an in-depth computational analysis of the flexible βFαG′-loop, and its role in enzyme catalysis and in ligand binding were elucidated. Thus, a multi-trajectory/-complex molecular dynamic (MD) strategy was followed, based on four different enzyme conformations. Molecular mechanics Poisson-Boltzmann surface area (MM-PBSA) methods [30], in combination with normal-mode analysis (NMODE) [31], were exploited to calculate the free energies of the complexes with the aim to substantiate our kinetic hypothesis and to determine the best enzyme conformation for binding of E1 and NADPH, respectively.

Moreover, we analyzed the kinetic mechanism of 17β-HSD1 by means of MD simulations in order to enlarge the conformational sampling of the enzyme structures. This ensemble of conformers should be used to improve drug-design through the identification of new inhibitor binding modes. In particular, making a parallel to *E. coli* DHFR which also has a bi-bi kinetics, our long range goal is to design 17β-HSD1 inhibitors able to “freeze” the enzyme in one of its kinetic substates, hindering the evolution of the kinetic cycle, in a similar way as methotrexate (MTX) does in DHFR [3].

## Results and Discussion

### 1. Structural analysis of human 17β-HSD1 crystal structures

17β-HSD1 is a homodimeric protein and shows the typical rigid β-α-β fold of the SDR-family with a core of parallel β-strands fanning across the center and α-helices draped on the outside (Figure S2) [19], resulting in a rigid COF at the N-terminus and a structurally variable C-terminus, hosting the SUB [32]. The structurally conserved region comprises Rossman fold and GxxxGxG motif (G is glycine and x any other residue), characteristic of oxidation/reduction enzymes that bind nicotinamide cofactors, plus the YxxxK sequence (Y is Tyr155 and K Lys159) that participates in catalysis [22]. In sharp contrast to other 17β-HSDs, type 1 is characterized by a long, flexible βFαG′-loop and a C-terminal helix, both delimiting the SUB and probably involved in enzymatic catalysis and in entrance and exit mechanisms of small molecules.

**Figure 3:**
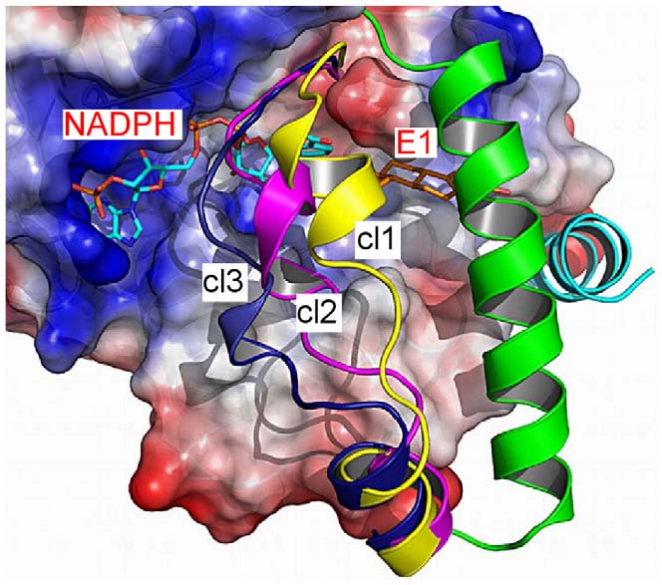
Clusters according to backbone RMSD. Representative structures of the three βFαG′-loop conformations cl1 (yellow, 1iol), cl2 (magenta, 1a27) and cl3 (blue, 1i5r), clustered according to the backbone RMSD of the five residues Phe192-Val196. βFαG′-loop, αG′-helix (green) and C-terminal helix (cyan) are rendered as cartoon, NADPH and E1 as sticks and the rest of the enzyme (active site) as electrostatic surface.

Until May 2010 twenty crystal structures of 17β-HSD1 were available in the Protein Data Bank (PDB) as apoform, binary or ternary complex (Table 1). The loop residues Thr190-Gly198 of the PBD entry 1fdt [37] were modelled in two different conformations and had been considered as two different conformers, denoted hereafter as 1fdtA and 1fdtB. Superimposition of all crystals gave a mean backbone RMSD (root mean-square distance) of ca. 0.5 Å (all-atom RMSD ca. 1.5 Å). The only flexible part was identified in the above mentioned βFαG′-loop, which could adopt different conformations, strongly modulating shape and volume of the active site (including both COF and SUB). Eight of these crystal structures were incomplete, missing mainly the loop residues Phe192-Val196, while the remaining eleven showed high b-factor values for this area, an additional hint for its flexibility. In the fully resolved structures the loop occurred as a disordered random coil. Moreover, the loop was found in a stable orientation only for ternary complexes, which had enzyme, cofactor and steroidal product or inhibitor fully resolved (1a27, 1fdtA, 1fdtB, 1equA, 1fduC and 3hb5; cluster cl2 in Figure 3). For all other crystal structures no univocal loop conformation could be ascribed to the presence of either cofactor or ligand. Nevertheless, this multiplicity of conformations suggested various substates for 17β-HSD1, which are subjected to fast dynamic motions and secondary structure rearrangements of the loop. Most of the binary complexes E-E2 were fully resolved, while the E-NAD(P)^+^ complexes lack part of the βFαG′-loop and no binary complex with NADPH or with E1 existed at all (Table 1). The existence of numerous fully resolved E-E2 complexes led us to speculate that NADPH became tightly bound to the enzyme only after the steroid had entered the cavity and the loop had changed its conformation. Interestingly, a similar behaviour was confirmed also by molecular dynamics of some complexes, as it will be elucidated later in this article.

**Table 1:** Summary of the 20 (21) crystal structures of 17β-HSD (April 2010).

PDB	Resid	ligands^a^	Res (Å)	Chain	backbone cluster of βFαG′-loop^b^	mutants	active site volume (Å^3^) calculated with castP[^33^]	
					(loop helix)		COF	SUB
1a27 [^34^]	285	E2, NAP	1.90	A	cl2	–	1855	
							1232/66%	623/34%
1bhs [^19^]	284	–	2.20	A	cl1	–	2214	
					A191-E194			
1dht [^35^]	284	DHT	2.24	A	cl1	–	1944	
					M193-V196			
1equ [^36^]	284	EQI, NAP	3.00	A/B	cl2 (A)/cl1 (B)	–	2403	
					M193-V196		1606/67%	797/33%
1fds [^37^]	282	E2	1.70	A	–	–	2323	
1fdtA [^37^]	285	E2, NAP	2.20	A	cl2	–	2000	
							1255/63%	745/37%
1fdtB [^37^]	285	E2, NAP	2.20	A	cl2	–	2259	
							1583/70%	676/30%
1fdu [^38^]	281	E2, NAP	2.70	A–D	cl2 (C)	H221L	C: 1645	
					M193-K195			
1fdv [^38^]	285	NAD	3.10	A–D	cl2 (A, C)	H221L	A: 1892/C: 2051	
					E194-V196			
1fdw [^38^]	279	E2	2.70	A	–	H221Q	2301	
1i5r [^39^]	285	HYC	1.60	A	cl3	–	2755	
1iol [^40^]	284	E2	2.30	A	cl1	–	2296	
					A191-V196			
1jtv [^41^]	278	TES	1.54	A	–	–	1855	
1qyv [^42^]	276	NAP	1.81	A	–	–	2906	
1qyw [^42^]	276	5SD, AP2	1.63	A	–	–	1858	
1qyx [^42^]	277	ASD, AP2	1.89	A	–	–	1888	
3dhe [^35^]	284	AND	2.30	A	cl1	–	2152	
					M193-V196			
3dey [^43^]	277	DHT	1.70	A	–	–	1882	
3hb4 [^44^]	284	E2B	2.0	X	cl2	–	2707	
3hb5 [^44^]	284	E2B, NAP	2.0	X	cl2	–	2179	
							1408/65%	771/35%
3klm [^45^]	277	DHT	1.70	A	–	–	1882	

a complexed ligands: E2 (estradiol), NAP (nicotinamide-adenine-dinucleotide phosphate; NADP^+^), DHT (dihydrotestosterone), EQI (equilin), NAD (nicotinamide-adenine-dinucleotide), HYC (O5′-[9-(3,17β-Dihydroxy-1,3,5(10)-estratrien-16β-yl)-nonanoyl] adenosine), TES (testosterone), 5SD (5α-androstane-3,17-dione), ASD (4-androstene-3,17-dione), AP2 (2′-monophosoadenosine 5′-diphosphoribose), AND (3β-Hydroxy-5-androstene-17-one).

b βFαG′-loop backbone clusters: (cl1) loop close to the αG′-helix, (cl2) loop placed in front of the nicotinamide moiety of the cofactor, (cl3) loop shifted towards the cofactor.

(**Resid**) total number of amino acids crystallized; (**Res**) resolution of the crystal structures in Å.

**Figure 4:**
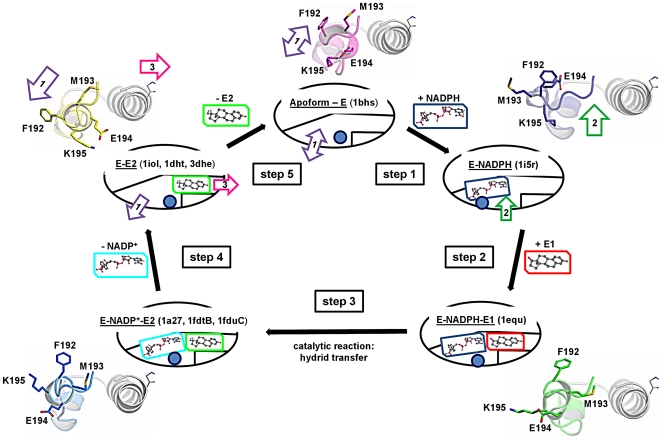
Random bi-bi kinetic cycle of 17β-HSD1. To each step one or more crystal structures were assigned. Side chains of the βFαG′-loop residues Phe192-Lys195 are rendered as sticks and the orientation of the loop (blue ball) with respect to the αG′ helix (in gray) is shown as cartoon. The arrows 1–3 indicate the entrance and egress gates identified along the reaction pathway. 1fdtA is not included in the balloon of **step 4** due to its different loop axis orientation with respect to 1iol.

The twelve full-length crystal structures of 17β-HSD1 were superimposed and clustered according to the backbone RMSD (mean value ca. 3 Å) of the five loop residues Phe192-Val196. Three clusters cl1, cl2 and cl3 (Figure 3; representative structures: 1iol, 1a27 and 1i5r, respectively) could be identified, as well as the presence of three gates, two close to the loop (gate 1 and 2) and a third one (gate 3) close to the C-terminal helix (Figure 4). However, this backbone classification disregarded active site volume changes and was limited to ascribe the presence of opened gates for the three clusters cl1–cl3. Thus, clustering was repeated taking into account all atoms of the same loop residues, which resulted in a mean RMSD value of ca. 6 Å. Five clusters could be identified (Figure 4 and S3) and Phe192, in particular, emerged as an important marker for the conformational variations, since its side chain presented the largest RMSD deviation (ca. 10 Å) rotating for about 200 degrees around the loop axis (Figure S4 and Video S1): it turned from the inner cavity, where it occluded the SUB and stabilized the substrate in the correct position for hydride transfer (1fdtB), toward the outside, where it chaperones E1/E2 or the cofactor in and out of the active site (1fdtA). These two extremes in the orientation of the side chain of Phe192 corresponded to the major changes of the active site volumes of all wild type E-NADPH-E2 complexes, where the volume ratio SUB/COF changed from 30∶70 in 1fdtB to 37∶63 in 1fdtA (Table 1).

### 2. Which mechanisms govern the kinetic cycle of 17β-HSD1?

17β-HSD1 is described to follow a random bi-bi kinetic mechanism (Figure 4, S1 and S3) [14], [16]. Based on biochemical and structural data (i.e. the excess of NADPH over E1 and the different crystal structures for the apo-, holo- and ternary form) we hypothesized 17β-HSD1 to follow a five-step preferential pathway *in vivo*. Moreover, we assigned enzyme conformations to the specific steps and tried to rationalize the reaction pathway according to the presence of cofactor, E1 or E2, as well as to the conformational changes expected to occur in order to allow the kinetic cycle to evolve further (in brackets the PDB id of the crystal structures best describing the starting conformer for each specific step).

#### 2.1. Step 1 (1bhs)

We postulate that NADPH will bind first and, in particular, into the COF, when the βFαG′-loop is placed in cluster cl2/1, with gate 1 wide and accessible. The entrance of the cofactor and its interactions with the apoform of the enzyme might induce a conformational rearrangement of the flexible loop toward the COF, with the loop moving from cl2 into cl3, thus closing gate 1 and opening gate 2. This loop-motion is intended to favour the entrance and binding of E1.

#### 2.2. Step 2 (1i5r)

E1 enters and the loop slides back to its starting point in cluster cl2/1, gate 2 will be closed again and the next step reached.

#### 2.3. Step 3 (1fdtB, 1a27)

After the βFαG′-loop has allowed the entrance of E1, it orients Phe192 and Met193 inward in order to stabilize the substrate in an optimal position for the *pro*-S hydride transfer. The first ternary complex (E-NADPH-E1) is formed and the catalytic reaction takes place.

#### 2.4. Step 4 (1fdtA, 1iol)

Now the flexible loop is expected to drift into cluster cl1, protruding more into the SUB and opening gate 1. This gate rules the egress path for NADP^+^, hence when it is open the oxidized cofactor can leave the active site. The steroid, however, is still stabilized in the SUB, shielded by Phe192 and Met193.

#### 2.5. Step 5 (no crystal structure given)

Herein the steroid leaves the active site through gate 3.

Interestingly, for all five substates the loop residues and Phe226 on the αG′-helix are oriented in a way to avoid solvent accessibility to the SUB.

### 3. Validation of the catalytic cycle by means of molecular dynamic simulations

MD simulations have been proven to be very useful tools in the analysis of dynamic motions of enzymes with flexible loops, which might play a prominent role in either the catalytic cycle and/or ligand binding. In particular, kinetic cycle and ligand-binding related motions of *E. coli* [1], [3] have been intensively studied by means of both experimental and computational methods. While the former (such as site-directed mutagenesis, isothermal calorimetry, NMR dispersion, crystallography, etc.) constituted the basis for the understanding of DHFR functionality and catalytic mechanism, the computational methods (such as MD simulations, thermal fluctuation, free energy calculations, etc.) were applied with success in order to elucidate protein plasticity and allosteric mechanism. Moreover, they were envisaged to address new strategies in drug design, as for example the inhibition of *E. coli* DHFR by functional disruption, where the enzyme is frozen in a conformer that stops the kinetic cycle [3].

Herein, we applied MD simulations and free energy calculations pursuing as a main goal to substantiate the assignment of different enzyme conformations of 17β-HSD1 to the five steps encountered along its catalytic pathway. Moreover, we wanted to investigate transition steps and enzyme motions influenced by starting conformation and ligand binding. To this end, eight MD simulations (models I-III; Text S1) were designed based on the crystal structures 1fdtA, 1i5r, 1fdtB, and 1bhs, which differ strongly in the orientation of the βFαG′-loop (Figure 3, 4 and S3). In fact, the enzyme was investigated in its opened-state (1fdtA) - characterized by a fused COF and SUB and by Phe192 turned outward, in a semi-opened/occluded state (1i5r) - with the loop shifted toward the COF and Phe192 more buried than in 1fdtA, and in its closed-state (1fdtB) - mimicking the catalytic moment with a closed SUB and Phe192 pointing inward perpendicular to the catalytic Tyr155. The MD simulations of apoform E (A), binary E-NADPH (B) and ternary complexes E-NADPH-E1 (C) of 17β-HSD1 were evaluated in terms of geometry (RMSD) and energy stability of the complexes, ligand and secondary structure displacement with respect to the starting pose, and the time required to reach a stable RMSD and energy plateau. For each stable sector of the MD trajectories absolute free energy (ΔG) and relative binding affinity (ΔG_bind_) were calculated by means of MM-PBSA methods and NMODE analysis. Thus, in order to determine whether a preferential enzyme conformation exists for the binding of NADPH and E1, we compared ΔG and ΔG_bind_ values of the binary complexes B1–B3 and of the ternary C1–C3, respectively (Table 2).

**Table 2:** Free energy calculations for the MD simulations B1–B3 and C1–C3.

E	PDB	MD code	ELEC	VDW	GAS	PBSOL	PBTOT (ΔG_bind_)	TSTOT	ΔG
			mean	mean	mean	mean	mean (±SE)	mean (±SE)	mean
Model II - **E-NADPH**									
opened	1fdtA	**B1**	−380.1	−42.87	−371.7	386.95	15.3±1.3	−20.2±3.1	35.5±2.2
occluded	1i5r	**B2**	−484.9	−70.11	−555	510.69	**−44.27±1.7**	−34.92±3.4	**−9.3±2.6**
closed	1fdtB	**B3**	−358.8	−74.54	−433.3	411.6	−21.69±0.9	−37.16±3.1	15.5±2
Model III - **E-NADPH-E1**									
opened	1fdtA	**C1**	0.35	−38.8	−38.58	18.12	−20.47±0.5	−17.87±3.4	−2.6±1.9
occluded	1i5r	**C2**	−12.72	−36.81	−49.53	25.88	−23.65±0.4	−22.69±3.1	−0.96±1.8
closed	1fdtB	**C3**	−23.43	−36.75	−60.29	31.99	**−28.3±0.3**	−19.99±3.2	**−8.31±1.7**

**ΔG** and **ΔG_bind_** values correspond to the longest stable RMSD plateau for each MD; i.e. the values for **C1** and **C3** correspond to the plateaux until ca. 10 ns, while the free energy values for the extended (+6/7 ns) section of the MDs are reported in the text.

(**E**) starting conformation of the enzyme; (**ΔG**) absolute free binding energy; (**ΔG_bind_**) (**PBTOT**) relative free binding energy; (**ELEC**) electrostatic contribution in gas phase; (**VDW**) Van der Waals contribution in gas phase; (**GAS**) free energy in vacuum; (**PBSOL**) solvation energy; (**TSTOT**) (**TΔS**) entropic contribution; (**mean**) mean value; (**SE**) standard error of the mean; all energies expressed in kcal/mol.

#### 3.1 Step 1

The MDs performed for **step 1** pursued two main goals. First, we wanted to identify the enzyme conformation best representing the enzyme in its apoform and, thus, the folding that the flexible βFαG′-loop will adopt in absence of other ligands. And second, we expected to find an easily accessible COF, and eventually SUB, in the stable parts of the trajectories, as a consequence of opened gates 1 and 2.

Two MD simulations were performed with the enzyme only (1bhs – A1, 1fdtB – A2; Figure S5). In the apoform crystal structure 1bhs (A1) the fully resolved βFαG′-loop laid in cluster cl1, presenting a turn-helix-turn motif and the side chains of its residues oriented along its axis. Thus, a 12 ns MD A1 was performed in order to verify the reliability of 1bhs as **step 1** enzyme conformation. Remarkably, the loop evolved toward cluster cl2, shielding the COF and especially the area where the nicotinamide moiety is found. This led to a large active site suitable to host both cofactor and ligand. The enzyme showed the tendency to preserve the hydrophobic character of the SUB, turning the side chains of the apolar residues (mostly Phe192, Phe226 and Phe259) inward, which occluded the SUB and avoided water to be placed in there. Met193 was also deeply buried in the SUB, occupying the space where steroids are normally placed. On the contrary, all polar residues belonging to the FG segment and to the C-terminal helix turned outside forming ion-pairs responsible for the closure of gate 2 and 3. The final stable complex of this MD was considered as a transition state of **step 1**, with an accessible COF, suitable to host NADPH, and an occluded SUB, not ready to accommodate the substrate. According to our hypothetic cycle, the βFαG′-loop should drift now further toward the COF (i.e. into cluster cl3), in concert and/or slightly delayed to NADPH entrance, resulting in a progressive closure of the COF (closed gate 1) and accessibility of the SUB (opening of gate2; 1i5r).

The second MD A2 was performed on the apoform of the closed-state conformation 1fdtB, stripped of both cofactor and steroid. The aim was to investigate whether the starting enzyme conformations of A1 and A2 would lead to different final tertiary structures or if they could converge to a common folding and loop axis orientation. However, already after 1 ns of simulation a marked change in the tertiary structure of the 1fdtB apoform was observed involving FG segment and C-terminal helix. Phe192 remained turned inward, while Met193 rotated out toward the αG′-helix, becoming responsible for the disruption of the αG′-helix.

The two simulations did not converge to a common conformation; moreover, the final complex of A2 resulted in a partially unfolded structure. This result suggested that the presence of cofactor and/or ligand would be necessary to assure a correct transition between the five steps of the enzymatic cycle and that the choice of the starting conformation might be crucial as well. The stable trajectory of A1, however, represents a nice simulation of **step 1**, especially if the exposed COF is considered.

#### 3.2. Step 2

In **step 2** the COF should already be occluded and gate 1 being closed by the motion of the flexible loop into cl3. The loop should stabilize NADPH in the COF, and also maintain the substrate in the SUB in a correct position for the hydride transfer. The latter requires the loop to float again into cluster cl2, thus centrally placed.

Three 9 ns MDs were carried out on the binary complex E-NADPH (B) and another three, lasting 10 ns, on the ternary complex E-NADPH-E1 (C), with the enzyme in its opened (1fdtA - B1, C1), semi-opened (1i5r - B2, C2) and closed (1fdtB - B3, C3) state, respectively. The aim was to obtain insights into the role played by the loop in the stabilization of NADPH, the shielding of the SUB and the stabilization of E1.

Simulation B2 (1i5r-NADPH) was characterized by a quickly reached, very long stable RMSD plateau (Figure S6), indicating a good stability of the complex. Also the loop showed a stable RMSD profile, although, while the lower part (Glu194-Ser199) still remained close to the cofactor in cluster cl3, the upper part (Thr190-Met193) drifted toward the αG′-helix. This seemed to occur as a direct consequence of the outward rotation of Phe226, which was responsible to sustain the nicotinamide moiety in the starting complex, but which was also involved in opening gate 2, a prerequisite for E1 entrance. The loop was folded to a short helix and stabilized by the ion-pair Lys195 and 2′-phosphate of the cofactor, which trapped the adenosine moiety. In the final structure the upper part of the loop, placed in cluster cl2, occluded part of gate 2, reducing the solvent accessible surface and preserving the hydrophobic character of the SUB. Nevertheless, a hydrophobic tunnel remained open between loop and helix, suitable for steroid transition. The complex stability of B2 was also reflected by a good free energy ΔG (−9.35 kcal/mol) with respect to the positive values of B1 and B3 (Table 2), which substantiated our structural hypothesis for catalytic **step 2**.

**Figure 5:**
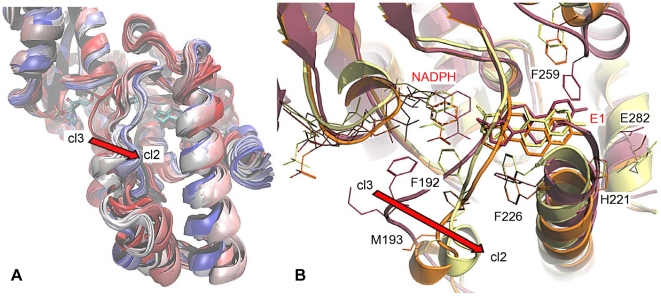
Motion of the βFαG′-loop in MD C2 (1i5r). (A) The overlay of multiple snapshots of the trajectory C2 showed the transition of the loop axis from cluster cl3 to cl2 (color-coded in a range from red to blue, according to start and end of the MD, respectively). (B) Important active site residues of the initial (violet, **step 1**), stable (orange, **step 2**) and final complex (yellow) are rendered as sticks.

The MD of the ternary complex 1i5r-NADPH-E1 (C2) described very well the expected loop motion from cluster cl3, shielding the COF, to cluster cl2 (Figure 5). Despite a suboptimal ΔG (−0.96 kcal/mol; but with a good ΔG_bind_ of −23.68 kcal/mol) compared to that of the closed-state complex C3 (−8.31 kcal/mol), we considered the MD C2 as a valuable simulation of **step 2** from a binary state to the ternary complex (Figure 5B). Effectively, this simulation ended with a stable complex in which E1 was properly bound into the SUB, involved into an h-bond network with Ser142/Tyr155 and His221/Glu282. Furthermore, the βFαG′-loop drifted into cl2 shielding the active site and forming a short α-helix, characteristic for most of the ternary complexes present in the PDB.

#### 3.3. Step 3

In this step the hydride transfer is meant to take place, thus no major tertiary structure rearrangements were expected.

The three MDs of the ternary complex E-NADPH-E1 (C1–C3; Figure S7) highlighted very well the important role of Phe192 and Met193 in stabilizing E1 close to the cofactor to ensure an optimal distance necessary for hydride transfer. When these two residues were turned outward, as it happened for the opened and the semi-opened complexes 1fdtA-NADPH-E1 (C1) and 1i5r-NADPH-E1 (C2) respectively, E1 had more space to move at the beginning of the simulations and resulted less stably bound.

The catalytic residues Ser142, Tyr155, and Lys159 showed small RMSD values for all three ternary complexes, even after 10 ns of dynamics (Figure S7D). This observation was in accordance to experimental site-directed mutagenesis studies [22], where the enzymatic activity was abolished because of the mutation of one or more of these residues. Moreover, the conformational stability of these residues suggested them to be essential for the postulated catalytic mechanism (Figure 2) [46], while other regions, for example the βFαG′-loop, to be the driving force that keeps the catalytic cycle running. The trajectories observed for the FG-segment residues Phe192 (in particular), Lys195 and Phe226 in the three simulations C1–C3 overlapped nicely with their orientations in the eighteen enzyme conformations (Figure S4) and fitted very well with the conclusion of Mazza et al. [37], who suggested a direct involvement of Lys195 and Phe192 in the catalytic cycle.

Phe192 was meant to play a very important role not only in steroid stabilization, but also in the catalytic reaction itself. In most ternary complexes with E1, Phe192 pointed perpendicular toward Tyr155. In this T-shaped conformation, Phe192 is likely to increase the acidity of the phenol group of the catalytic Tyr155 from a pK_a_ of ca 9 to 6 [47], which presumably might facilitate the hydride transfer to the estrone. The final pose of Phe192 in C2 and C3 was consistent with this observation and enforced its role in protecting the nicotinamide moiety and activating the catalysis.

#### 3.4. Postulated entrance mechanism of E1

**Figure 6:**
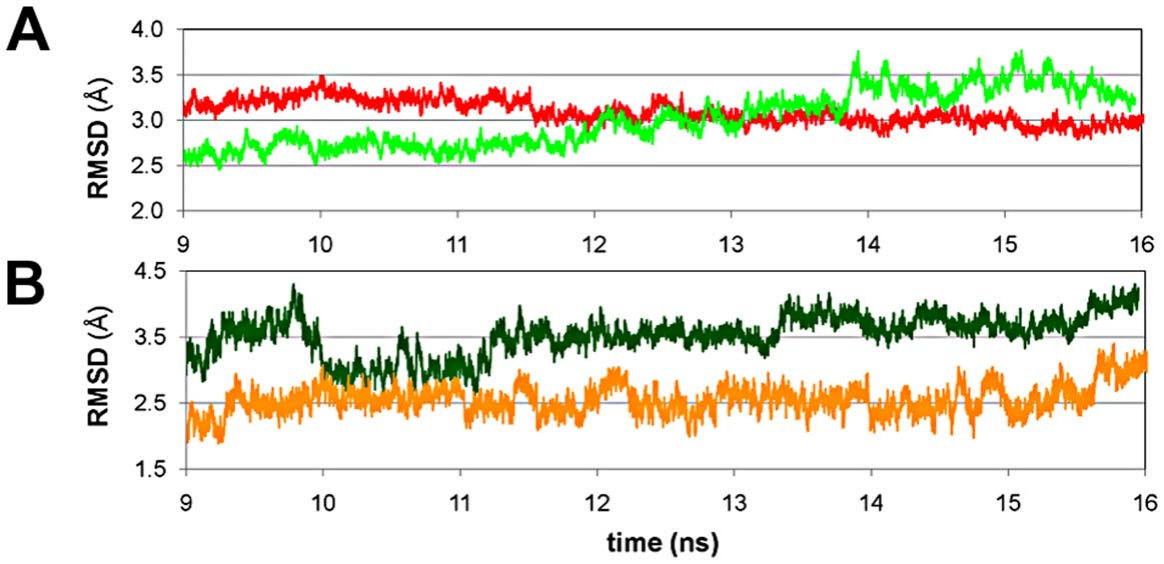
RMSD analysis of the last 7 ns of MDs C1 and C3. (A) Time-dependent all-atom RMSD for all residues of the ternary complexes C1 (1fdtA) (light green) and C3 (1fdtB) (red) and (B) for the βFαG′-loop residues 187–199 of C1 (dark green) and C3 (orange).

**Figure 7:**
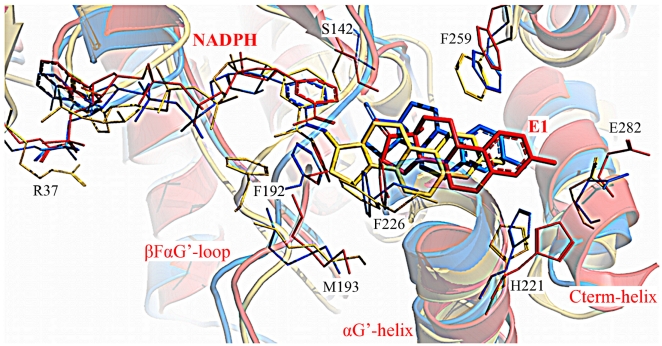
Representative snapshots of the MD C3. Overlay of the representative structures of the initial position (yellow), the stable segment (blue, **step 3**) and the extended part (red, **step 5**) of the MD C3 (1fdtB).

The starting structures of the opened and the closed complexes C1 and C3 (1fdtB-NADPH-E1), respectively, presented the loop axis (main chain) in similar starting conformation (cluster cl2), but with the side chains of the loop residues oriented opposite. After ca. 9 ns the MD C1 presented a long, stable RMSD plateau (Figure S7B). However, when this simulation was prolonged to ca. 16.5 ns, the all-atom RMSD rose to 3.5 Å, mostly related to βFαG′-loop motions (Figure 6). In fact, the extended part of C1 was characterized by slow concerted motions of the FG-segment and the C-terminal helix, i.e. increasing loop RMSD in Figure 6B, which led to the opening of gate 2 (the loop moves toward the COF into cl3) and gate 3. Remarkably, concomitant to these rearrangements the ΔG increased from −2.6 (after 9 ns) to −6.02 kcal/mol (after 16.5 ns). A progressive loss of the hydrogen bond between E1 and Ser142 was observed in concert with the outward rotation of Met193, Phe192, and Phe226. Thus, E1 drifted beneath the cofactor, turning its D-ring toward gate 2. A “backward analysis” of the whole simulation C1, ending with a conformation similar to the starting one of C3 (1fdtB), led us to postulate a “pull-and-push” mechanism for E1 entrance (Figure S8): E1 enters the binding site through gate 2, pulled by the inward rotation of Lys195 to which the steroid is bound with the C17 carbonyl. This bond will be released once E1 is placed into the SUB and Lys195 could rotate further toward the 2′-phosphate of the cofactor to stabilize it. Thus, Phe192 turns inward drifting below the nicotinamide ring of NADPH pushing hereafter E1 into the SUB and reaching so a similar conformation as the starting structure of C3 (1fdtB-NADPH-E1). Other residues delimiting gate 2 might also adapt to the steroid, as for example Phe226, which turns from the outside to the inner cavity stabilizing E1 (stable complex of MD C3; in blue in Figure 7).

#### 3.5. Step 4

In step 4 we expected the βFαG′-loop to drift further toward the αG′-helix into cluster cl1, protruding slightly into the SUB and opening gate 1, and, as a direct consequence, that NADP(H) leaves the COF via gate 1.

Two out of the six binary and ternary MD simulations were meaningful for describing this step: 1fdtB-NADPH-E1 (C3), which confirmed the loop motion with subsequent opening of gate 1 and the drift of E2 toward gate 3; and 1fdtA-NADPH (B1), which showed the egress path of the cofactor due to gate 1 enlargement.

Although at this stage the steroid should be E2, we considered for **step 4** the simulations C1–C3, where E1 and NADPH were used instead of E2 and NADP^+^. We consider this approximation as acceptable, because no differences between the hydrogen bond patterns of E1 and E2 with 17β-HSD1 exist and because forcefield based methods, such as MD simulation, are not sensitive enough to handle the electrostatic differences between ligand and cofactor forms [25]. Moreover, in this study we focused on the role of βFαG′-loop residues in substrate binding and on the determination of protein-ligand binding thermodynamics, and not on the simulation of the hydride transfer in the E1-E2 reduction. For the latter high-level quantum chemical calculations would be required, capable of dealing with bond-formation and –breaking as well as of treating electrons explicitly, in contrast to force field-based methods which handle atoms. In all complexes, E1 was placed in the same pose, analogous to that of E2 in the crystals. As already seen, all the MDs C1–C3 reached a stable RMSD plateau after already 4 ns (Figure S7), maintaining E1 placed in the SUB, but the ΔG values for their stable segments varied notably (Table 2). The MD of the ternary complex C3 best described **step 4** with an ΔG of −8.31 kcal/mol for the first 10 ns (ΔG_bind_ −28.3 kcal/mol): E1 maintained its hydrogen bonds to Ser142/Tyr155 for the first 10 ns, until being pushed toward gate 3 and out of the SUB in the following 7 ns due to the loop motion toward the αG′-helix (Figure 7) and the inward rotation of Phe192 and Phe226.

On the other hand, in simulation 1fdtA-NADPH (B1), characterized at its beginning by a wide access to the COF due to the outward rotated Phe192, we observed a concerted opening of gate 1 and closing of gate 2 due to the shift of the loop axis toward the αG′-helix. Thus, after a gradual increase in the first 5 ns, the all-atom RMSD reached a plateau and maintained it until the end of the simulation. Differently, the cofactor RMSD continued to rise (Figure S6D), in accordance to the movement of NADPH out of the COF, which was facilitated by the outward rotated Arg37, not burying the adenosine moiety in the COF anymore. Notably, the cofactor-stabilizing role of Arg37 had been previously demonstrated by site-directed mutagenesis studies [18]. The αG′-helix rotated slightly rightward for about 30 degrees, adapting its residues to those of the loop. In this motion, His221 moved outward, breaking the salt bridge with Glu282 and enlarging gate 3. The free energy of complex B1 (+35.53 kcal/mol) clearly indicates that this enzyme conformation is disfavoured to stabilize NADPH, which ends up solvent exposed, suggesting complex B1 to be a good model to simulate the exit of the cofactor in **step 4**.

#### 3.6. Step 5

At this stage we expected E2 (in our case E1) to exit the SUB via gate 3 as well as a concerted rearrangement of the FG-segment residues to shield the empty SUB.

MD simulation C3 was prolonged up to ca 17.5 ns, with the purpose to evaluate the propensity of E1 to escape the active site. While the all-atom RMSD of the extended part of C3 (10–17.5 ns) decreased (Figure 6A), its loop RMSD increased slowly (Figure 6B). This sector of the dynamic was characterized by the closure of gate 2, the opening of gate 3 and the concerted breaking of the hydrogen-bond network between E1, His221 and Glu282. In fact, in C3 E1 drifted toward gate 3 and stuck out of the SUB. The progression of E1 to the end of the tunnel was favoured by the loop motions, the rotation of the apolar residues Phe192, Met193 and Phe226 into the SUB (Figure 7), and by the edging out of His221 and Glu282 and the consequent enlargement of gate 3. Interestingly, a decrease of ΔG from −8.31 (after ca. 10 ns) to −6.36 kcal/mol (after 17 ns) was observed.

Ideally, the final structure of **step 5** should be similar to the starting one of **step 1**. This could not be reached with our 10 ns MDs. The reasons therefore could be the presence of E1 in the SUB, influencing the loop motions, but also because a much longer simulation time would be required to sample such marked conformational changes. However, the final structure of C3 reveals opened gates 1 and 3 and an occluded gate 2, with the βFαG′-loop close to the αG′-helix, as observed for 1bhs, the representative crystal for **step 1**.

Very recently, a binary and a ternary complex of 17β-HSD1 (PDB entry 3hb4 and 3hb5) with the potent E2-derived inhibitor E2B were published [44]. In the ternary structure the βFαG′-loop was oriented in cluster cl2, in analogy to all other ternary complexes, confirming its importance in ligand stabilization, in particular of Phe192 and Met193.

The results of the analysis of the crystal structures and of the multi-trajectory MD approach coupled with free energy calculations were in agreement with the biochemical data published so far and substantiated the random bi-bi kinetic cycle of 17β-HSD1. Our multi-trajectory approach allowed an accurate assignment of different enzyme conformers to each postulated step of the catalytic cycle of 17β-HSD1. The analysis of the different trajectories revealed an essential role for backbone and side chain motions, in particular of the flexible βFαG′-loop, in cofactor and substrate binding, and the existence of distinguished conformer ensembles related to the single steps of the catalysis. Thus, it suggested 17β-HSD1 to follow a selected-fit mechanism [48]: E1 or NADPH binds to a transition state of the enzyme and induces concerted conformational rearrangements of FG-segment and C-terminal part, which then guides the enzyme along its preferred catalytic pathway. Furthermore, the enzyme-ligand (both NADPH and E1) motions observed in our MD study suggested a preferential pathway for this kinetic cycle, with the cofactor NADPH entering first, followed by the substrate E1, which then induces concerted enzyme adaptation to both ligands. Depending on the presence of E1 and/or NADPH these enzyme forms evolved into a favourable low energy conformations representative for the specific steps, as for example observed for the ternary complex 1I5R-NADPH-E1 (C2) where the flexible loop moved toward the αG′-helix stabilizing both the substrate E1 and the cofactor NADPH.

The small RMSD variations observed for the catalytic residues Ser142, Tyr155 and Lys159 in all MDs suggested other regions of the enzyme to be responsible for the proceeding of the five-step catalytic cycle. Site-directed mutagenesis studies indicated for His221 and Glu282 a prominent role in substrate recognition, but not in catalysis [34], [36]–[39]. The motions of His221 observed in the simulations were in agreement with these results and allowed interesting conclusions regarding the role of His221 in substrate recognition and with respect to its chaperone function in guiding E1 out of the SUB (Figure 7).

Ghosh *et al.* described two conformations for the βFαG′-loop (“substrate-entry loop”), an opened when no steroids are present and closed one with the steroid in the SUB [36], [49]. On the contrary, in our study we identified three main βFαG′-loop axis and five different loop side-chains orientations, characteristic for five steps of the bi-bi kinetic cycle. Our multi-trajectory/-complex approach allowed us to sample a broad conformational space and all these conformers, together with the calculated ΔG values, substantiated the three cluster variant, with the enzyme in an opened (cl1), occluded (cl3) and closed (cl2) state, depending on the presence of steroid, cofactor-steroid or cofactor.

In conclusion, this study laid the basis not only for a better understanding of the kinetic cycle of 17β-HSD1 and of the role of some amino acids, in particular of Phe192, Met193, Lys195 and Phe226, but also for possible new design strategies to inhibit 17β-HSD1. Thus, the enzyme conformations assigned to the various steps might represent a valid starting point for the development of inhibitors disrupting the enzyme dynamics and thus inactivating it by freezing it in one conformer and avoiding the cycle to evolve further. However, direct experimental results are still needed for a final demonstration, and, in particular, site directed mutagenesis studies, as well as further computational investigations, are envisaged in order to strengthen our conclusions.

## Materials and Methods

### 1. Computational Details

Crystal structures of 17β-HSD1 were obtained from the Protein Databank (PDB, www.pdb.org) [50] and further prepared using the BIOPOLYMER module of SYBYL v8.0 (Sybyl, Tripos Inc., St. Louis, Missouri, USA). In detail, we used the following PDB structures: 1a27, 1bhs, 1dht, 1equ, 1fds, 1fdtx2, 1fdu, 1fdv, 1fdw, 1i5r, 1iol, 1jtv, 1qyv, 1qyw, 1qyx, 3dhe, 3dey, 3hb4 and 3hb5. 1fdt presents Arg37 and the loop residues Thr190 to Gly198 in two very different conformations, denoted in this article as 1fdtA (open state) and 1fdtB (closed state). Water molecules, sulfate ions and the hydride inhibitor HYC (1i5r) were removed from the PDB files and missing protein atoms were added. E2 was modified to estrone E1 and NADP^+^ was turned into NADPH. Hydrogen atoms and neutral end groups were added. All basic and acidic residues were considered protonated and deprotonated, respectively. Histidines oriented toward the outer part of the enzyme were considered as protonated after a prediction run made by MolProbity [51]. For 1i5r the cofactor NADPH was merged into the enzyme after an accurate overlay with the hybrid inhibitor HYC and the X-rays 1a27 and 1fdt. Further, every crystal structure was minimized for 500 steps with the steepest descent minimizer as implemented in SYBYL with the backbone atoms kept at fixed positions in order to fix close contacts, followed by 2000 steps conjugate gradient minimization requested for an overall better starting structure.

Ligands were described with the general Amber force field GAFF [52]. RESP charges for estrone were calculated, while the parameters of Ulf Ryde were taken for NADPH (charge -4) (http://www.teokem.lu.se/~ulf/).

The clustering of the crystal structures of Table 1 has been performed by means of the CONSENSUS utility of the homology module of MOE (www.chemcomp.com) and resulted in the two different backbone and side-chain classifications.

### 2. MD simulations

Molecular dynamics (MD) and free energy computations were carried out using Amber 9.0 suite of programs [53] and the AMBER99SB force field [54]. The simulation system was set up using the xLeap program of the AMBER suite. The simulation systems were surrounded by a truncated octahedral box of TIP-3 water molecules of 10 Å radius. Counterions were added to neutralize the system. Prior to the free MD simulations, the simulation systems were energy minimized for 5000 steps of steepest descent followed by 10000 steps of conjugate gradient optimization. The equilibration process was carried out with the program PMEMD on the NVT ensemble using the following procedure: the simulation system was heated during 200 ps from 0 to 200 K at constant volume conditions. This temperature was held for additional 200 ps and afterwards raised to 300 K during 200 ps at constant volume. Protein heavy atoms, NADPH and E1 were constraint, and the force constant was gradually reduced from 100 kcal/mol/Å^2^ to 2 kcal/mol/Å^2^. The final coordinates of the temperature equilibration routine were relaxed without restraint for other 50 ps and then used for the MD production run, performed at NPT physical conditions and without restraints. The total simulation length differed for the various complexes ranging from 9 to 17.5 ns. taup = 2.0 and cut = 10.0. Temperature regulation was done at 1.0 atm of pressure (1 atm = 101.3 kPa) by using a Langevin thermostat with a coupling constant of tautp = 1.5 ps. The time step of the free MD simulations was 2 fs, with a cutoff of 13 Å for the nonbonded interaction, and SHAKE [55] was employed to keep all bonds involving hydrogen atoms rigid. Electrostatic interactions were computed using the Particle Mesh Ewald method [56]. All simulations were carried out in periodic boundary conditions.

The analysis of the trajectories of the MD simulations was performed with the PTRAJ module of AMBER, the MMTSB tool set (http://mmtsb.org) [57] and using visual molecular dynamics (VMD) [58].

### 3. Free energy calculations using the MM/PBSA method

Conventional MM-PBSA [30], [59] and normal-mode (NMODE) [31] calculations were performed using the AMBER 9 suite. The electronic and Van der Waals energies were calculated by the *sander* module. The polar solvation energy was calculated with the finite-difference PB equation solver by using AMBER toolset.

For each stable sector of the MD trajectories B1–B3 and C1–C3, longer than 4 ns, snapshots were collected every 30th frame (every 30 ps) and used to calculate relative binding affinity (ΔG_bind_) and absolute free energy (ΔG) by means of MM-PBSA methods and NMODE. The normal mode analysis was performed to estimate the vibrational component of the entropy. Conjugate gradient and then Newton-Raphson minimizations until the root mean square of the elements of gradient vector was less than 5*10^−5^ kcal/mol were carried out in absence of solvent. A distance-dependent dielectric constant was used to mimic solvent screening. Frequencies of the vibrational modes were computed at 300 K for these minimized structures and using a harmonic approximation of the energies. Due to the high computational demand, only snapshots taken every 100^th^ frame from MD were used to estimate -TΔS.

A more detailed description of MM/PBSA and NMODE is given in Text S2.

### 4. RESP charges

The *ab-initio* geometry optimizations for E1 was performed in gas phase at the B3LYP/6-311+g (d,p) level of density functional theory (DFT) by means of the Gaussian03 [60].

## Supporting Information

Text S1 A detailed description of the multi-trajectory/-complex MD approach, classified as three models.
(0.10 MB DOC)

Text S2 Additional informations to the methods (MM-PBSA and NMODE) used.
(0.09 MB DOC)

Figure S1 Random sequential bi-bi kinetic cycle of 17β-HSD1. The preferred pathway is represented in red, guided by the excess of NADPH compared to E1, and identified by the MD simulations A1–A2, B1–B3, and C1–C3. *In vitro*, where the concentrations of the reagents can be modified, different orders might also be possible.
(0.33 MB TIF)

Figure S2 Tertiary structure of 17β-HSD1. Rossmann fold, βFαG′-loop, αG′-helix are highlighted. E2 and NADP+ are rendered as sticks, whereas the enzyme as cartoons. Helices are colored red, β-sheets in yellow and coils in green). The active site surface is rendered as grey wireframes. (PDB entry 1a27.)
(0.74 MB TIF)

Figure S3 Schematic representation of the catalytic cycle of 17β-HSD1. (A) 2D-scheme of the structure of 17β-HSD1; including: Rossmann fold, cofactor binding site (COF; blue), substrate active site (SUB; green), alternative (fused COF-SUB) active site, αG′-helix (green ball), βFαG′-loop (yellow-red ball); gates 1 (blue), 2 (cyan) and 3 (magenta) are represented by arrows and e circles of the same color. (B–F) Representative conformations of the 5 steps of the catalytic cycle clustered accordingly to the side-chain RMSD. Detailed informations about the role of the βFαG′-loop in the various steps of the cycle are mentioned in the single charts. The circles represent gates 1–3, and they change in size depending whether they are opened or closed. The loop residues Phe192 (F), Met193 (M), Glu194 (E) and Lys195 (K) are schematized according to their effective distance from the loop axis at every step.
(4.90 MB TIF)

Figure S4 Morphing the transition from 1fdtA to 1fdtB. Different orientations of Phe192 for the 5 clusters obtained by all-atom RMSD classification of the five loop residues (rendered in sticks, color-coded blue-violet) and for the 17 intermediate positions (rendered in lines, magenta) obtained by simulating the transition from 1fdtA to 1fdtB, the two extremes in the Phe192 rotation, using the Yale Morph Server (Krebs WG, Gerstein M (2000). The morph server: a standardized system for analyzing and visualizing macromolecular motions in a database framework. Nucl Acids Res 28:1665–1675). A movie of this rotation is also available (Video S1).
(2.07 MB TIF)

Figure S5 Analysis of the MDs of the apoform complexes A1–A2. Time-dependent all-atom RMSD for (A) all residues of the complexes A1 (blue - 1bhs) and A2 (red - 1fdtB), and (B) only for the βFαG′-loop residues only. (C) Residue-dependent RMSD fluctuation for the three complexes A1–A2.
(3.69 MB TIF)

Figure S6 Analysis of the MDs of the binary complexes B1–B3. Time-dependent all-atom RMSD for the complexes (A) B2 (light blue all residues, dark blue βFαG′-loop), (B) B1 (light green all residues, dark green βFαG′-loop) and (C) B3 (red all residues, orange βFαG′-loop). (D) Time-dependent RMSD for NADPH of the three complexes B2 (blue), B1 (green) and B3 (red). (E) Residue-dependent RMSD fluctuation for the three complexes B1–B3 (same colors as for (D)).
(5.22 MB TIF)

Figure S7 Analysis of the MDs of the ternary complexes C1–C3. (A) Time-dependent all-atom RMSD for the complexes C2 (light blue all residues, dark blue βFαG′-loop), (B) C1 (light green all residues, dark green βFαG′-loop) and (C) C2 (red all residues, orange βFαG′-loop). (D) Residue-dependent RMSD fluctuation for the three complexes C1–C3.
(4.89 MB TIF)

Figure S8 Postulated entrance mechanism for E1. (A) Four snapshots of the MD C1 (1fdtA-NADPH-E1), with representative structures after (Ai) 12 ns (white), (Aii) 9 ns (yellow), (Aiii) 6 ns (magenta) and (Aiv) 3 ns (magenta). Surfaces of the ligand binding sites are shown in light grey. (B) Starting structure (violet) and stable complex after 10 ns (cyan) of MD C3 (1fdtB-NADPH-E1). Complexes are rendered in cartoon. NADPH, E1 and residues crucial for the dynamic are shown as sticks and labeled.
(3.91 MB TIF)

Video S1 Morphing the transition from 1fdtA to 1fdtB.
(2.61 MB MPG)
