# Supplementary material for: Insights in 17β-HSD1 Enzyme Kinetics and Ligand Binding by Dynamic Motion Investigation
Source: PLoS One. 2010 Aug 10;5(8):e12026. doi: 10.1371/journal.pone.0012026 (PMC2919385; doi:10.1371/journal.pone.0012026)
Supplement: Text S1 — A detailed description of the multi-trajectory/-complex MD approach, classified as three models. (0.10 MB DOC) [file pone.0012026.s001.doc]

### **Molecular Dynamic simulations – 3 models:**

For model I two MD simulations were performed on the apoform of the enzyme (1bhs – A1, 1fdtB – A2). 1bhs is the only resolved apoform deposited in the PDB and the side chains of the loop residues are oriented along the loop axis. This leads to a large active site, with gates 1 and 2 opened, suitable to host both cofactor and ligand. Goal of this last model was to judge the influence of the absence of both cofactor and ligand on the ternary structure and on the dynamic behaviour of 17β-HSD1. In model II three MD simulations were conducted on the binary complex E-NADPH, with the enzyme in its opened (B1), semi-opened (B2) and closed (B3) state, respectively, in order to obtain insights in the role played by the loop in the stabilization of NADPH and in the shielding of the SUB. Furthermore, we wanted to substantiate our choice of crystal structure 1i5r as probable **step 2** in the kinetic cycle by evaluating the propensity of NADPH to exit the COF depending on the loop orientation. In model III the focus is on the binding of E1and three MDs are performed on the E-NADPH-E1 complex of each state (C1-C3).

**Model I – apoform of 17β-HSD1.** A long MD simulation A1 (ca. 12 ns) has been performed for 1bhs, with a fully resolved loop in cluster cl1 with the turn-helix-turn motif, in order to verify its reliability as **step 1** enzyme conformation (Fig. 4 and S3). Remarkably, the βFαG’-loop evolves toward cluster cl2, shading partially the COF, especially where the nicotinamide moiety is located. The enzyme shows the tendency to preserve the hydrophobic character of the SUB, turning the side chains of almost all hydrophobic residues inward, especially of the aromatic residues, which occlude the SUB and avoid water to be placed in there. Met193 reaches deeply in the SUB, occupying the space where steroids are normally placed. On the contrary, all polar residues belonging to the F/G segment and the C-terminal helixes are turned outside forming ion-pairs, responsible for the closure of gate 2 and 3. The final stable complex presents an accessible COF, suitable to host NADPH, and an occluded SUB, not ready to accommodate the substrate.

In order to substantiate the conformation of the apoform, a second MD (A2) was conducted on the transition-state like conformation 1fdtB, stripped of both cofactor and steroid. Already after ca. 1 ns a marked change in the ternary structure is observed involving F/G segment and C-terminal helix. Phe192 remains turned inward, but Met193 rotates toward the αG’-helix, as observed for B3, becoming the main responsible for the helix distortion. This movement results in a less shielded SUB, due to hindered formation of ion-pairs between loop and αG’-helix and an opened gate 2.

**Model II – role of the βFαG’-loop in stabilizing the cofactor.** Three MD simulations (B1-B3) have been performed, each of ca. 9 ns length, and evaluated accordingly to the solvent exposure of the cofactor as a consequence of the motions of the βFαG’-loop and its neighboring structural motifs.

In the MD simulation B1 we observe the progressive opening of gate 1 and the concomitant disappearing of gate 2 due to the shift of the loop axis toward the αG’-helix. Thus, after a gradual increase of ca. 5 ns the RMSD reaches a plateau, maintained until the end of the simulation, while the cofactor shows rising RMSD values, confirming its motion out of the COF. The αG’-helix rotates slightly rightwards for about 30°, adapting its residues to that of the loop. His221 is edged outward, breaking the salt bridge with Glu282 and enlarging gate 3. The free energy calculated for the B1 (+35.53 kcal/mol) clearly indicates this enzyme conformation as disfavored to stabilizing the NADPH, which ends up distorted and solvent exposed**,** suggesting complex B1 to be a better model to simulate **step 4** of the kinetic cycle (Fig. 4).

On the other hand, the simulation B2 is characterized by a fast reached, very long (ca. 8 ns) stable RMSD plateau, indicative for a good overall stability of the complex. This fits for the loop as well, although while the lower part (Glu194-Ser199) still remains close to the cofactor in cluster cl3, the upper part (Thr190-Met193) drifts toward the αG’helix. We see herein a direct consequence of the outward rotation of Phe226, responsible to sustain the nicotinamide moiety in the starting complex, facilitated by the opened gate 2. The nicotinamide ring drops down and becomes supported by the inward rotated Phe192, but at a lower level, thus lying farer away from Val188, which was responsible for a stabilizing hydrogen bond with the amide function of NADPH [5]. The loss of this interaction is compensated by the ability of the loop to fold to a short helix and by the ion-pair between Lys195 and the 2’-phosphate of the cofactor, which stabilizes the adenosine moiety. In the final structure the upper part of the loop, placed in cluster cl2, occludes part of gate 2, reducing the solvent accessible surface and preserving the hydrophobic character of the SUB. Nevertheless, a hydrophobic tunnel remained between loop and helix, suitable for steroid transition. The complex stability is reflected also by a good free energy value (-9.35 kcal/mol), which substantiates our structural hypothesis for catalytic **step 2**.

B3 evolves to a similar final structure as obtained for complex B2, especially regarding the lower part of the loop. Lys195 and the 2’-phosphate are involved in ion-pair formation, while Phe192, already turned inward at the beginning, stabilizes the nicotinamide moiety. As a consequence of the loop rearrangement, Met193 is turned toward the αG’-helix, inducing a helix distortion and pushing Phe226 out of the active site. This conformational changes does not influence the C-terminal part, leaving gate 3 opened. The ΔG for this complex B3 is +16.61 kcal/mol, but this positive value results mainly from the huge entropic contribution, as a consequence of helix distortion and loop movement.

**Model III - validation of estrone binding and conclusions regard the catalytic cycle**. In the three MD simulations performed for model I (C1-C3) E1 occupies the same starting pose, close to that of E2 in the crystals. All the simulations reach a fairly stable RMSD plateau after ca. 4 ns, maintaining it for at least other 6 ns and with E1 still placed in the SUB (Fig. 5, 7 and S8). Nevertheless, the ΔG values for the stable segments range from -8.31 kcal/mol of complex B3 to -2.6 and -0.96 kcal/mol of complex C1 and C2, respectively. Notably, these values are strongly influenced by different entropy contributions (Tab. 2) and complex C3, which is formed with crystal structure 1fdtB and which we assigned to **step 3** of the kinetic cycle, results in the best ΔG due to reduced conformational rearrangements and a low entropy term.

This set of MDs highlights very well the important role of Met193 and Phe192 in stabilizing E1 close to the cofactor ensuring an optimal distance (ca. 3.5 Å) necessary for hydride transfer, as seen for many crystal structures. When they are turned outward, as for complex C1 and C2, E1 has more space and is less stable bound.

### **SI References:**

1. Shi R, Lin SX (2004). Cofactor hydrogen bonding onto the protein main chain is conserved in the short chain dehydrogenase/reductase family and contributes to nicotinamide orientation. J Biol Chem 279:16778-16785.
