# Supplementary material for: Insights in 17β-HSD1 Enzyme Kinetics and Ligand Binding by Dynamic Motion Investigation
Source: PLoS One. 2010 Aug 10;5(8):e12026. doi: 10.1371/journal.pone.0012026 (PMC2919385; doi:10.1371/journal.pone.0012026)
Supplement: Text S2 — Additional informations to the methods (MM-PBSA and NMODE) used. (0.09 MB DOC) [file pone.0012026.s002.doc]

**MM-PBSA and NMODE.**

Conventional MM-PBSA [1-2] and normal-mode (NMODE) [3] calculations were performed using the AMBER 9 suite [4]. The electronic and Van der Waals energies were calculated by the Sander module.

The binding free energy resulting from the formation of the protein-ligand complex is approximated by the following equation:

ΔG =ΔH – TΔS 1)

in which *T* is the temperature of the system at 300 Kelvin.

The binding free energy (Δ*G*) of the protein–ligand complex resulted from:

ΔG = G_complex_ – (G_protein_ + G_ligand_) 2)

where *G*_complex_ is the absolute free energy of the complex, *G*_protein_ is the absolute free energy of the protein, and *G*_ligand_ is the absolute free energy of the ligand.

For each species (complex, protein, and ligand) snapshots were extracted from all stable sectors of the trajectories lasting at least 4 ns every 30^th^ step.

The enthalpy term in eq 1 consists of following subenergy terms:

H_tot_ = H_gas_ + G_solv_ 3)

H_gas_ = E_elec_ + E_vdw_ + E_int_ 4)

where *H*_gas_ is the potential energy of the solute, *E*_vdw_ the sum of van der Waals energy, *E_elec_* the electrostatic energy and *E*_int_ the internal energies in gas phase by using the SANDER module of Amber. *G*_solv_ is the solvation free energy and is given by the sum of electrostatic (*G*_elec_) and nonelectrostatic (hydrophobic) contributions (*G*_nonel_):

G_solv_ = G_elec_ +G_nonel_ 5)

The polar solvation energy was calculated with the finite-difference PB equation solver by using AMBER toolset. A spherical solvent probe (radii) of 1.4 Å and atomic radii provided by the Amber force field were used for the implicit solvent molecules and solute atoms, respectively, during the PBSA computations.

The absolute entropy was computed for each solute species by NMODE module of Amber 9.0. The total entropy (*S*_tot_) resulted from changes in the degree of freedom [translational (*S*_trans_), rotational (*S*_rot_), and vibrational (*S*_vib_)] of each species.

S_tot_ = S_trans_ + S_rot_ + S_vib_ 6)

Parameter/topology files used in MM-PBSA computations were prepared for the complex, the protein, and the inhibitors using the LEAP module. Snapshots extracted from trajectories were preminimized in the gas phase by the SANDER module using a conjugate gradient method until the root-mean-square-deviation of the elements of the gradient vector was less than 10^−4^ kcal/mol^−1^Å^−1^.

### **SI References:**

1. Kollman PA, et al. (2000) Calculating structures and free energies of complex molecules: combining molecular mechanics and continuum models. Acc Chem Res 33:889-897.
2. Srinivasan J, Cheatham TE III, Cieplak P, Kollman PA, Case DA (1998) Continuum solvent studies of the stability of DNA, RNA, and phosphoramidate-DNA helices. *J Am Chem Soc* 120:9401–9409.
3. Case DA, (1999) in Rigidity Theory and Applications, eds Thorpe MF, Duxbury PM (Plenum, New York), pp 329-344.
4. Case DA, Darden TA, Cheatham TE, Simmerling CL, Wang J, et al. Amber 9, University of California, San Francisco 2006.
