# Supplementary figures and images for: Insights in 17β-HSD1 Enzyme Kinetics and Ligand Binding by Dynamic Motion Investigation
Source: PLoS One. 2010 Aug 10;5(8):e12026. doi: 10.1371/journal.pone.0012026 (PMC2919385; doi:10.1371/journal.pone.0012026)

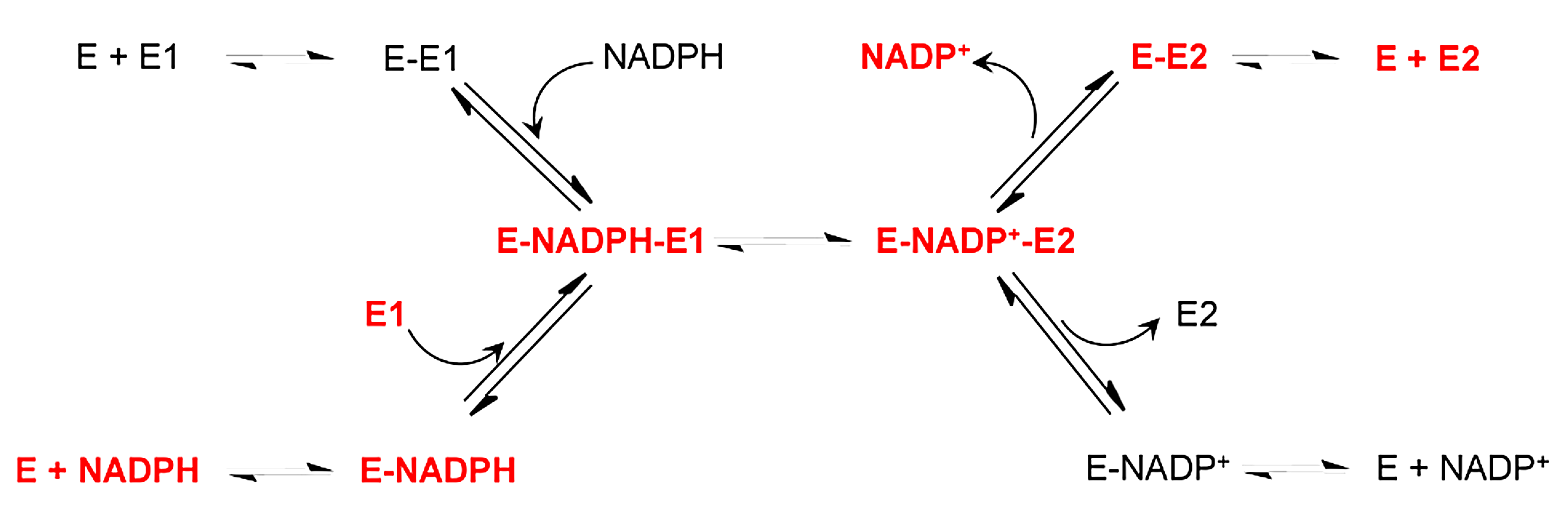

Supplement: Figure S1 — Random sequential bi-bi kinetic cycle of 17β-HSD1. The preferred pathway is represented in red, guided by the excess of NADPH compared to E1, and identified by the MD simulations A1–A2, B1–B3, and C1–C3. In vitro, where the concentrations of the reagents can be modified, different orders might also be possible. (0.33 MB TIF) [file pone.0012026.s003.tif]

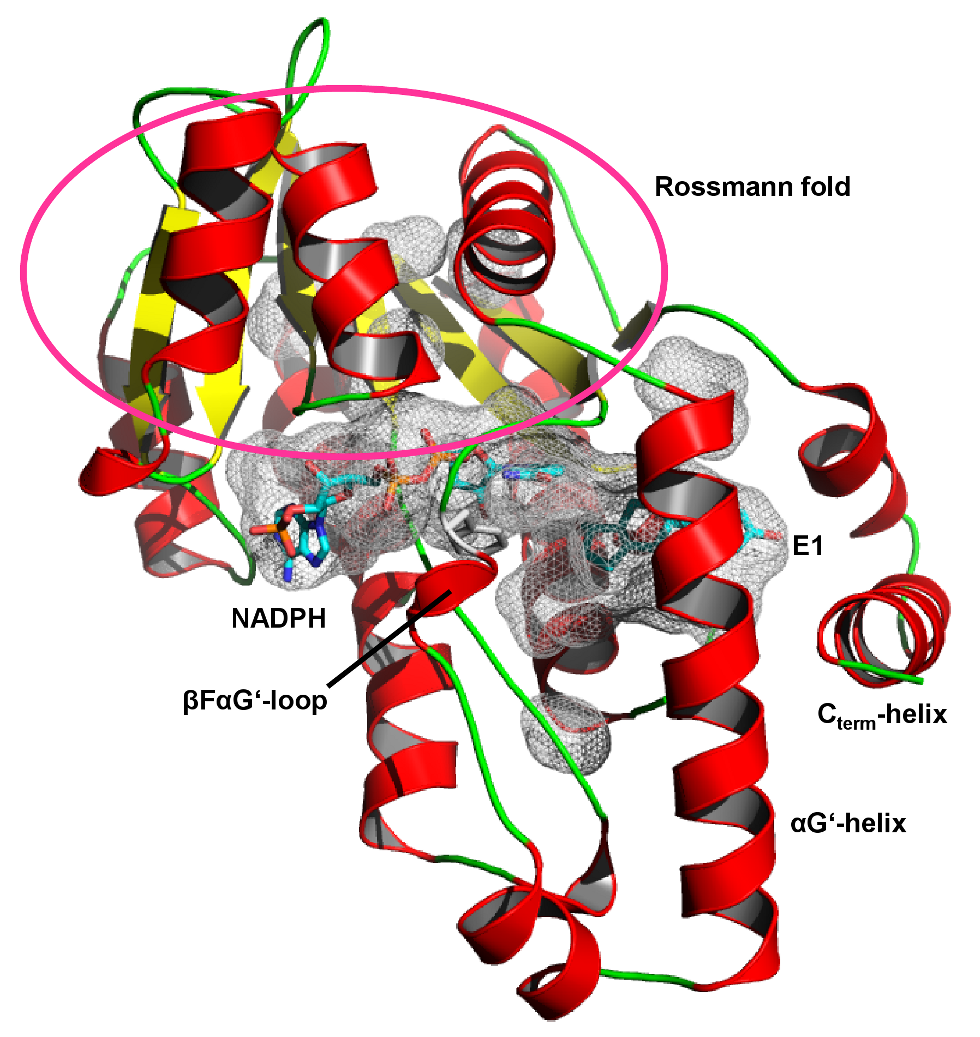

Supplement: Figure S2 — Tertiary structure of 17β-HSD1. Rossmann fold, βFαG′-loop, αG′-helix are highlighted. E2 and NADP+ are rendered as sticks, whereas the enzyme as cartoons. Helices are colored red, β-sheets in yellow and coils in green). The active site surface is rendered as grey wireframes. (PDB entry 1a27.) (0.74 MB TIF) [file pone.0012026.s004.tif]

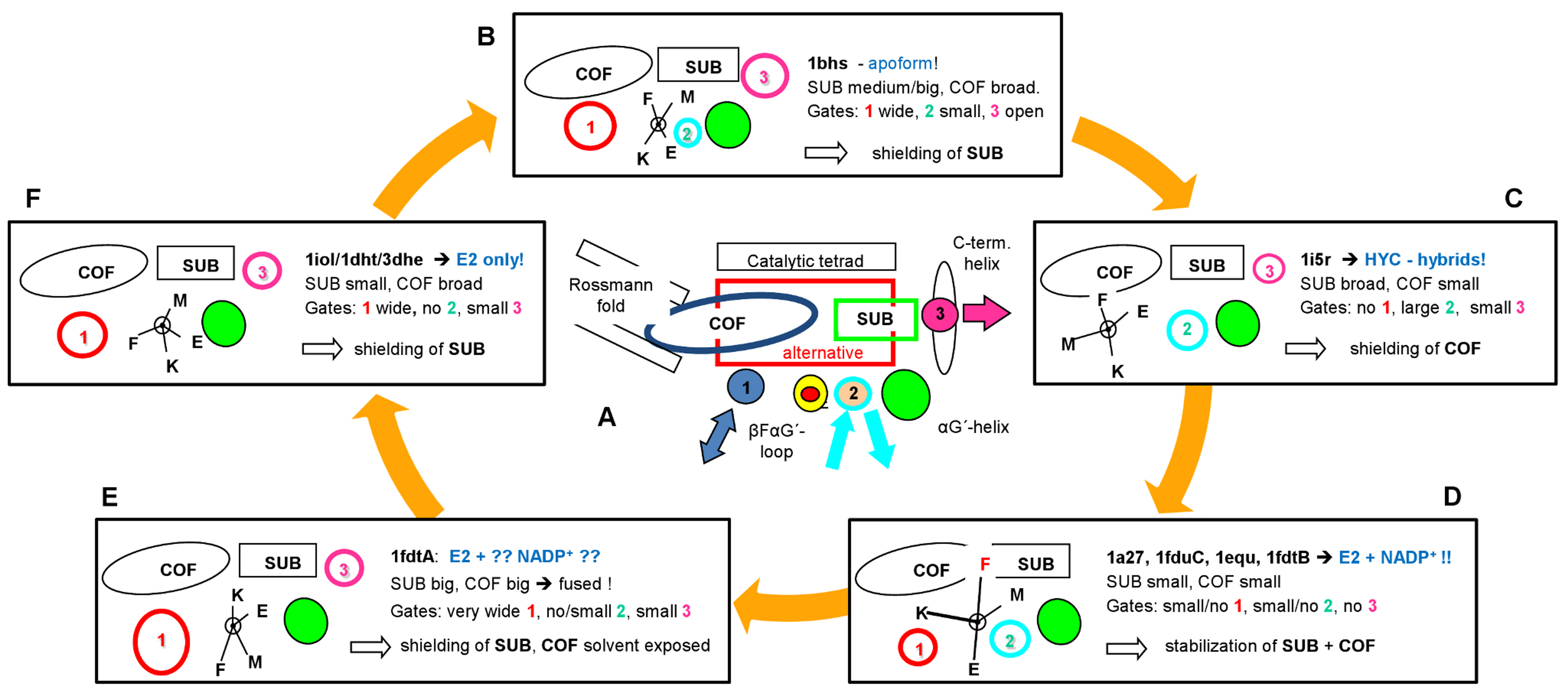

Supplement: Figure S3 — Schematic representation of the catalytic cycle of 17β-HSD1. (A) 2D-scheme of the structure of 17β-HSD1; including: Rossmann fold, cofactor binding site (COF; blue), substrate active site (SUB; green), alternative (fused COF-SUB) active site, αG′-helix (green ball), βFαG′-loop (yellow-red ball); gates 1 (blue), 2 (cyan) and 3 (magenta) are represented by arrows and e circles of the same color. (B–F) Representative conformations of the 5 steps of the catalytic cycle clustered accordingly to the side-chain RMSD. Detailed informations about the role of the βFαG′-loop in the various steps of the cycle are mentioned in the single charts. The circles represent gates 1–3, and they change in size depending whether they are opened or closed. The loop residues Phe192 (F), Met193 (M), Glu194 (E) and Lys195 (K) are schematized according to their effective distance from the loop axis at every step. (4.90 MB TIF) [file pone.0012026.s005.tif]

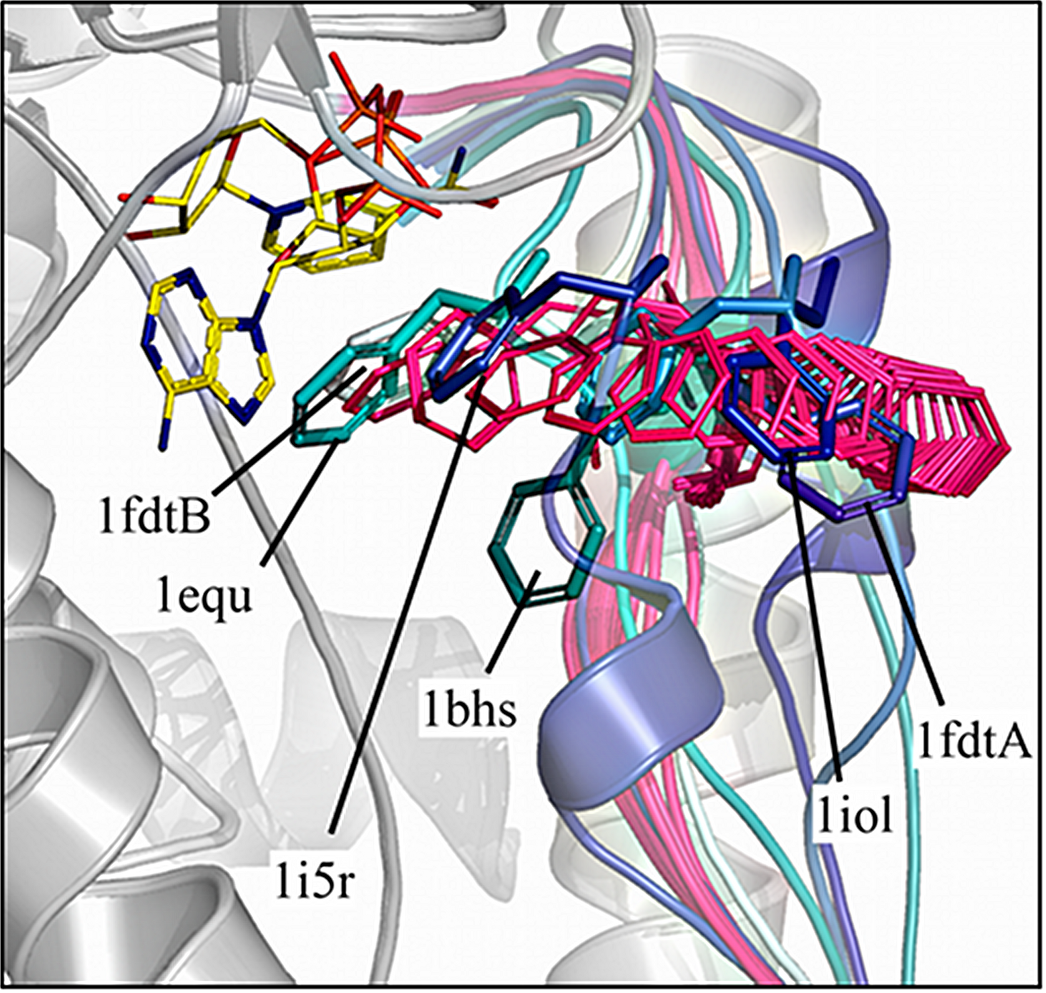

Supplement: Figure S4 — Morphing the transition from 1fdtA to 1fdtB. Different orientations of Phe192 for the 5 clusters obtained by all-atom RMSD classification of the five loop residues (rendered in sticks, color-coded blue-violet) and for the 17 intermediate positions (rendered in lines, magenta) obtained by simulating the transition from 1fdtA to 1fdtB, the two extremes in the Phe192 rotation, using the Yale Morph Server (Krebs WG, Gerstein M (2000). The morph server: a standardized system for analyzing and visualizing macromolecular motions in a database framework. Nucl Acids Res 28:1665–1675). A movie of this rotation is also available (Video S1). (2.07 MB TIF) [file pone.0012026.s006.tif]

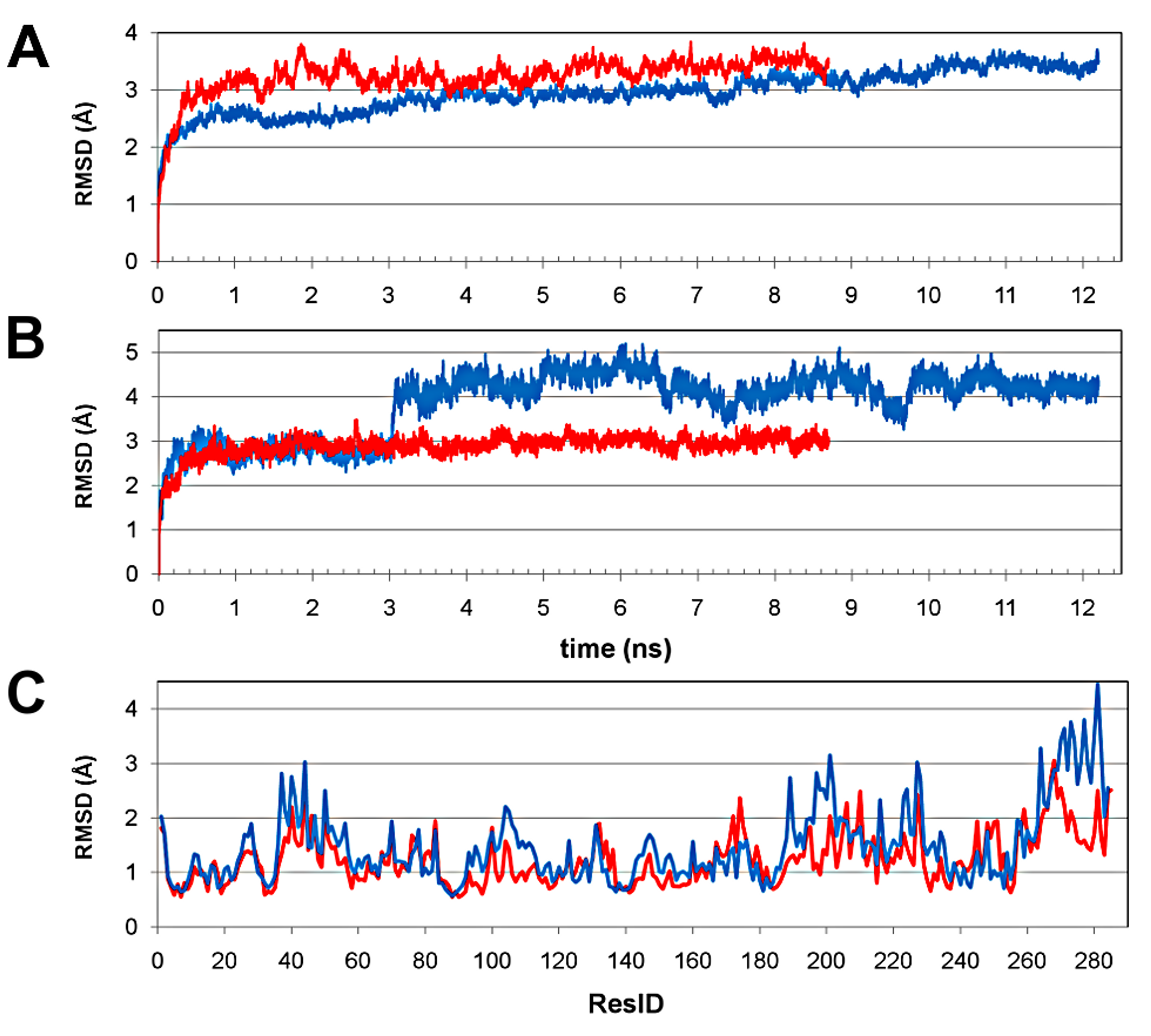

Supplement: Figure S5 — Analysis of the MDs of the apoform complexes A1–A2. Time-dependent all-atom RMSD for (A) all residues of the complexes A1 (blue - 1bhs) and A2 (red - 1fdtB), and (B) only for the βFαG′-loop residues only. (C) Residue-dependent RMSD fluctuation for the three complexes A1–A2. (3.69 MB TIF) [file pone.0012026.s007.tif]

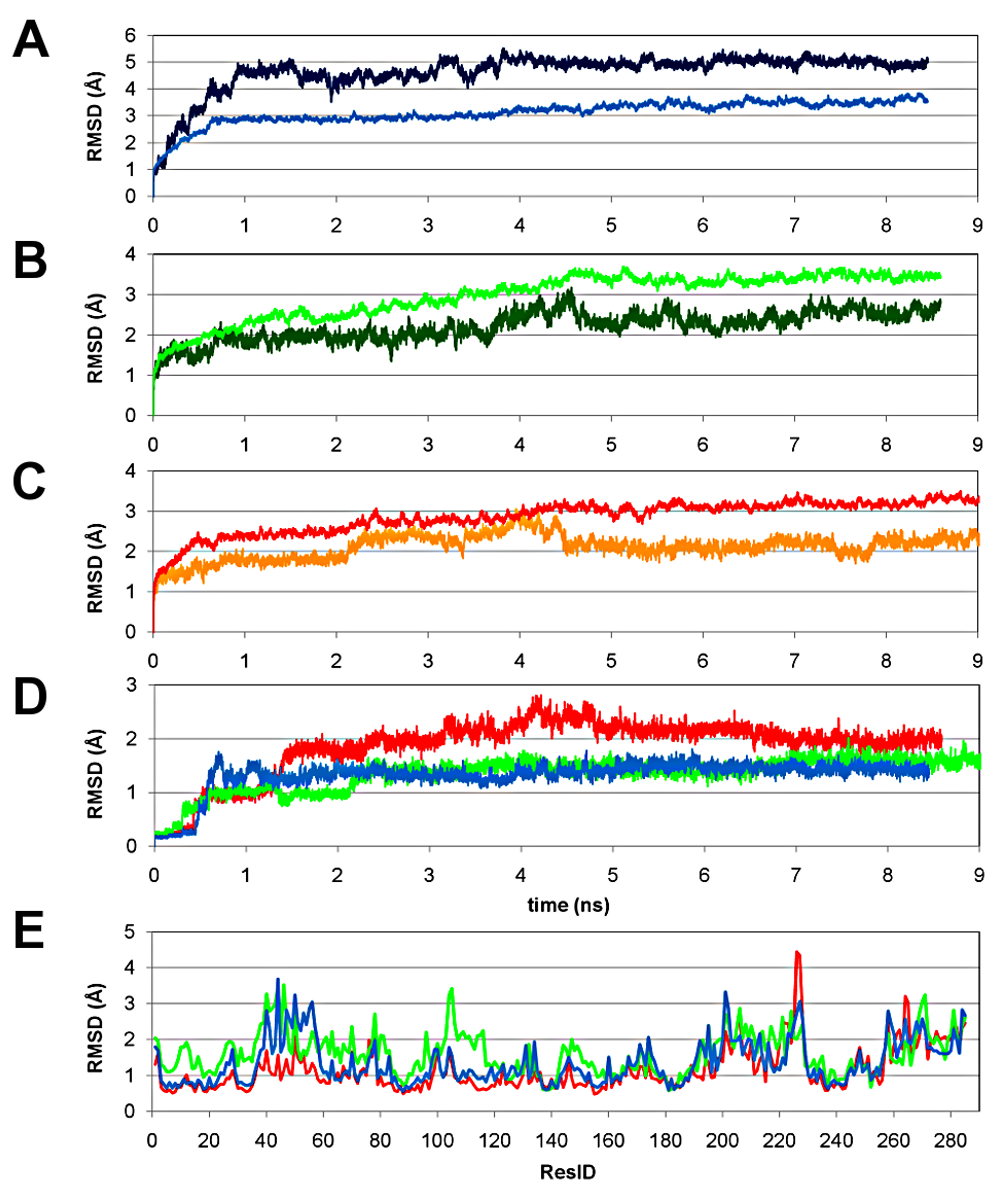

Supplement: Figure S6 — Analysis of the MDs of the binary complexes B1–B3. Time-dependent all-atom RMSD for the complexes (A) B2 (light blue all residues, dark blue βFαG′-loop), (B) B1 (light green all residues, dark green βFαG′-loop) and (C) B3 (red all residues, orange βFαG′-loop). (D) Time-dependent RMSD for NADPH of the three complexes B2 (blue), B1 (green) and B3 (red). (E) Residue-dependent RMSD fluctuation for the three complexes B1–B3 (same colors as for (D)). (5.22 MB TIF) [file pone.0012026.s008.tif]

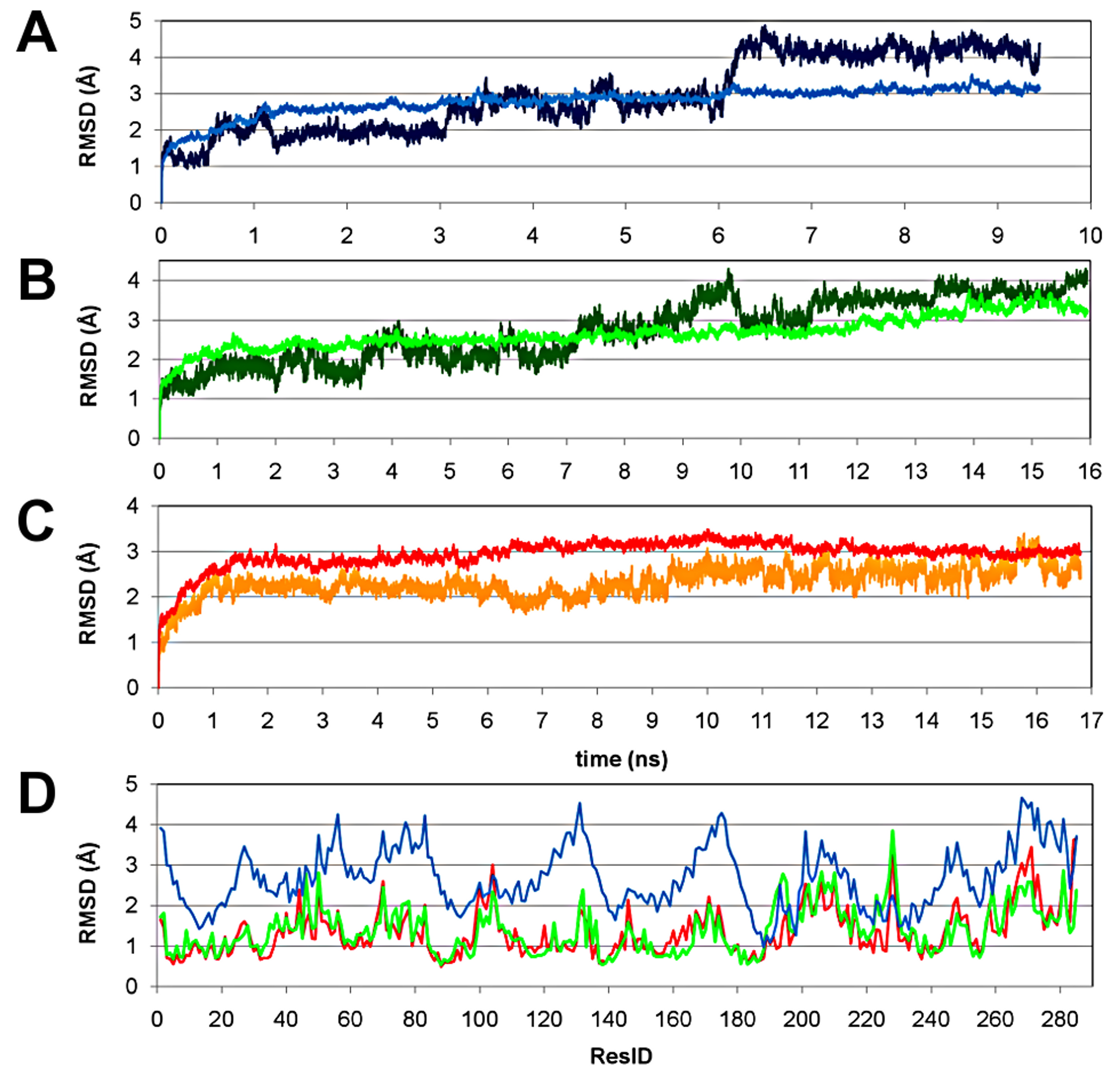

Supplement: Figure S7 — Analysis of the MDs of the ternary complexes C1–C3. (A) Time-dependent all-atom RMSD for the complexes C2 (light blue all residues, dark blue βFαG′-loop), (B) C1 (light green all residues, dark green βFαG′-loop) and (C) C2 (red all residues, orange βFαG′-loop). (D) Residue-dependent RMSD fluctuation for the three complexes C1–C3. (4.89 MB TIF) [file pone.0012026.s009.tif]

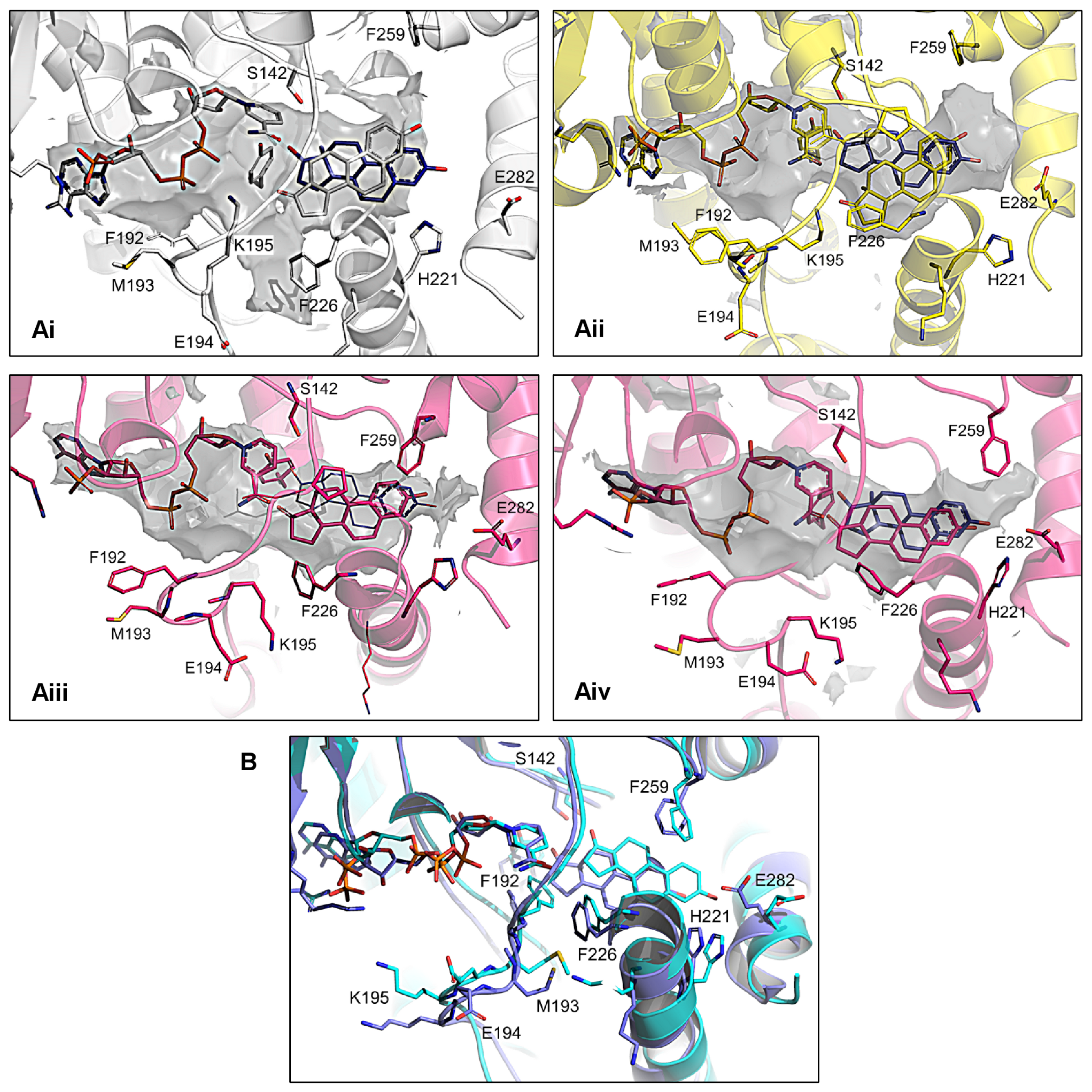

Supplement: Figure S8 — Postulated entrance mechanism for E1. (A) Four snapshots of the MD C1 (1fdtA-NADPH-E1), with representative structures after (Ai) 12 ns (white), (Aii) 9 ns (yellow), (Aiii) 6 ns (magenta) and (Aiv) 3 ns (magenta). Surfaces of the ligand binding sites are shown in light grey. (B) Starting structure (violet) and stable complex after 10 ns (cyan) of MD C3 (1fdtB-NADPH-E1). Complexes are rendered in cartoon. NADPH, E1 and residues crucial for the dynamic are shown as sticks and labeled. (3.91 MB TIF) [file pone.0012026.s010.tif]
